# Comprehensive Investigation on the Interplay between Feline APOBEC3Z3 Proteins and Feline Immunodeficiency Virus Vif Proteins

**DOI:** 10.1128/JVI.00178-21

**Published:** 2021-06-10

**Authors:** Yusuke Kosugi, Keiya Uriu, Narumi Suzuki, Keisuke Yamamoto, Shumpei Nagaoka, Izumi Kimura, Yoriyuki Konno, Hirofumi Aso, Brian J. Willett, Tomoko Kobayashi, Yoshio Koyanagi, Mahoko Takahashi Ueda, Jumpei Ito, Kei Sato

**Affiliations:** a Laboratory of Systems Virology, Institute for Frontier Life and Medical Sciences, Kyoto University, Kyoto, Japan; b Graduate School of Pharmaceutical Sciences, Kyoto University, Kyoto, Japan; c Division of Systems Virology, Department of Infectious Disease Control, International Research Center for Infectious Diseases, Institute of Medical Science, the University of Tokyo, Tokyo, Japan; d Graduate School of Medicine, the University of Tokyo, Tokyo, Japan; e Department of Molecular Virology, Tokyo Medical and Dental University, Tokyo, Japan; f Graduate School of Medicine, Kyoto University, Kyoto, Japan; g Graduate School of Biostudies, Kyoto University, Kyoto, Japan; h MRC-University of Glasgow Centre for Virus Research, University of Glasgow, Glasgow, United Kingdom; i Department of Animal Science, Faculty of Agriculture, Tokyo University of Agriculture, Kanagawa, Japan; j Department of Genomic Function and Diversity, Medical Research Institute, Tokyo Medical and Dental University, Tokyo, Japan; k CREST, Japan Science and Technology Agency, Saitama, Japan; Ulm University Medical Center

**Keywords:** virus evolution, FIV, Vif, APOBEC3, cross-species transmission, APOBEC3

## Abstract

As the hosts of lentiviruses, almost 40 species of felids (family *Felidae*) are distributed around the world, and more than 20 feline species test positive for feline immunodeficiency virus (FIV), a lineage of lentiviruses. These observations suggest that FIVs globally infected a variety of feline species through multiple cross-species transmission events during a million-year history. Cellular restriction factors potentially inhibit lentiviral replication and limit cross-species lentiviral transmission, and cellular APOBEC3 deaminases are known as a potent restriction factor. In contrast, lentiviruses have evolutionary-acquired viral infectivity factor (Vif) to neutralize the APOBEC3-mediated antiviral effect. Because the APOBEC3-Vif interaction is strictly specific for viruses and their hosts, a comprehensive investigation focusing on Vif-APOBEC3 interplay can provide clues that will elucidate the roles of this virus-host interplay on cross-species transmission of lentiviruses. Here, we performed a comprehensive investigation with 144 patterns of a round robin test using 18 feline *APOBEC3Z3* genes, an antiviral *APOBEC3* gene in felid, and 8 FIV Vifs and derived a matrix showing the interplay between feline APOBEC3Z3 and FIV Vif. We particularly focused on the interplay between the APOBEC3Z3 of three felids (domestic cat, ocelot, and Asian golden cat) and an FIV Vif (strain Petaluma), and revealed that residues 65 and 66 of the APOBEC3Z3 protein of multiple felids are responsible for the counteraction triggered by FIV Petaluma Vif. Altogether, our findings can be a clue to elucidate not only the scenarios of the cross-species transmissions of FIVs in felids but also the evolutionary interaction between mammals and lentiviruses.

**IMPORTANCE** Most of the emergences of new virus infections originate from the cross-species transmission of viruses. The fact that some virus infections are strictly specific for the host species indicates that certain “species barriers” in the hosts restrict cross-species jump of viruses, while viruses have evolutionary acquired their own “arms” to overcome/antagonize/neutralize these hurdles. Therefore, understanding of the molecular mechanism leading to successful cross-species viral transmission is crucial for considering the menus of the emergence of novel pathogenic viruses. In the field of retrovirology, APOBEC3-Vif interaction is a well-studied example of the battles between hosts and viruses. Here, we determined the sequences of 11 novel feline *APOBEC3Z3* genes and demonstrated that all 18 different feline APOBEC3Z3 proteins tested exhibit anti-feline immunodeficiency virus (FIV) activity. Our comprehensive investigation focusing on the interplay between feline APOBEC3 and FIV Vif can be a clue to elucidate the scenarios of the cross-species transmissions of FIVs in felids.

## INTRODUCTION

During the long history of the coevolution of viruses and their hosts, new lineages of viruses can emerge through cross-species viral transmission (1). To hamper viral cross-species transmission, host species have acquired intrinsic immunity during evolution (reviewed in references 2 to 4). One of the well-studied intrinsic antiviral factors that restricts viral replication in the host and potentially blocks viral cross-species transmission is apolipoprotein B mRNA-editing enzyme catalytic polypeptide-like 3G (APOBEC3G [A3G]) (5). Human A3G is a member of the APOBEC3 (A3) cytidine deaminase family of proteins and potently inhibits the replication of human immunodeficiency virus (HIV) type 1 (HIV-1), a lentivirus that causes AIDS in humans (reviewed in references 6 and 7). A3 proteins, including A3G, are incorporated into nascent HIV-1 virions and insert G-to-A hypermutations in newly synthesized viral genomes, resulting in the termination of viral replication. To eliminate the antiviral effect of A3 proteins, an HIV-1 accessory protein, viral infectivity factor (Vif), degrades the antiviral A3 proteins expressed in virus-producing cells via the ubiquitin/proteasome pathway (reviewed in references 6 and 8).

In terms of the roles of Vif and antiviral A3 proteins in cross-species transmission of lentiviruses, Zhang et al. have revealed that human A3H protein can act as a barrier that potentially impairs the cross-species transmission of lentiviruses from chimpanzees to humans (9). Additionally, we have shown recently evidence suggesting that gorilla A3G protein potentially contributes to the restriction of cross-species transmission of lentiviruses from chimpanzees to gorillas (10). Similarly, other studies have demonstrated the interplays between simian immunodeficiency viruses (SIVs) and primates (11, 12) and bovine lentiviruses and bovids (13).

The genus *Lentivirus* includes HIVs in humans, SIVs in nonhuman primates, bovine immunodeficiency virus and Jembrana disease virus in bovids, equine infectious anemia virus in horses, caprine arthritis encephalitis virus in goats, maedi-visna virus in sheep, and feline immunodeficiency viruses (FIVs) in felids (reviewed in references 3 and 14). As the hosts of lentiviruses, felids are most widely distributed in the world. Felids (i.e., the family *Felidae*) are estimated to have emerged around 10.8 million years ago (MYA), and almost 40 species of felids are distributed around the world, with the exception of Oceania (15).

Previous studies have shown that the interplay between FIV Vif and antiviral feline A3 proteins, particularly, APOBEC3Z3 (A3Z3) protein, is relatively unique compared to those between the other lentiviral Vif proteins and their host A3 proteins. First, de Castro et al. revealed that the *A3Z3* gene of domestic cats is polymorphic and that there are at least seven haplotypes (16). The *A3Z3* polymorphism in domestic cats is reminiscent of the findings showing the polymorphism of human *A3H*, the ortholog of feline *A3Z3* (17–21). In the case of human *A3H* haplotypes, some haplotypes (e.g., haplotypes II, V, and VII) express A3H proteins and exhibit anti-HIV-1 activity, while the others (e.g., haplotypes III, IV, and VI) do not express proteins because of the loss of protein stability (17–19, 21). On the other hand, we have previously shown that all seven domestic cat *A3Z3* haplotypes express proteins and exhibit anti-FIV activity (22). Interestingly, we demonstrated that a haplotype of domestic cat A3Z3, hap V, is resistant to the degradation mediated by all FIVfca (a class of FIV infecting domestic cat [Felis catus]) Vif proteins tested, suggesting the existence of domestic cat populations that are naturally resistant to FIVfca infection (22). Second, Zielonka et al. showed that FIVfca Vif can degrade not only the A3 proteins of its host, domestic cat, but also those of the other felids such as puma, lynx, lion, and tiger (23). On the other hand, we have reported that the Vif protein of FIVfca subtype B cannot degrade the antiviral A3 proteins of its host (24). Because FIVfca subtype B seems to be less pathogenic and divergent than the other FIVfca subtypes (25, 26), it is assumed that the loss of Vif’s ability to counteract antiviral A3 protein may be a way for FIVfca to adapt to the host through the attenuation of its virulence (24).

On the viral side, Troyer et al. have conducted a comprehensive surveillance for FIV infections on 35 feline species and revealed that at least 23 feline species tested were positive for either anti-FIV antibodies or FIV nucleotides (27). This comprehensive study indicates that FIVs globally infect a wide variety of host species. Specifically, the *vif* sequences have been determined in 5 FIV lineages in 5 felids: FIVfca in domestic cats (Felis catus), Pallas’s cats (Otocolobus manul; FIVoma), pumas (Puma concolor; FIVpco), bobcats (Lynx rufus; FIVlru), and lions (Panthera leo; FIVple) (28–32). In investigating the origin of HIV, previous studies showed SIV infection in a variety of Old World monkey species as well as nonhuman great apes (reviewed in reference 33). However, the habitat of the primates infected with SIVs is limited to Africa. In sharp contrast, FIVs are distributed globally and have been isolated from felids of multiple genera. Two felids, puma and bobcat, naturally live in North America, and interestingly, two different classes of FIVs, FIVpco and FIVlru, circulate between these two felids belonging to different genera (28, 34, 35). More intriguingly, we have previously demonstrated that all FIVlru Vifs tested can degrade both puma and bobcat A3Z3s, while a subclass of FIVpco Vifs can degrade puma A3Z3 but not bobcat A3Z3, suggesting that bobcat A3Z3 can potentially be a “species barrier” that hampers the cross-species transmission of a subclass of FIVpco from pumas to bobcats (36).

Several classes of FIVs and their host felids are distributed globally. Therefore, a comprehensive investigation focusing on the interplay between FIV Vif proteins and feline A3Z3 proteins may provide clues that will elucidate the roles of the Vif-A3 interplay on cross-species transmission of lentiviruses and the coevolution of lentiviruses and mammals. In this study, we newly determine the feline *A3Z3* sequences of 11 species and investigate the anti-FIV activities of the A3Z3 proteins of 18 felid species. We also determine a novel FIV *vif* sequence from leopard (Panthera pardus; FIVppa) and comprehensively test the ability of the 8 different FIV Vif proteins from 6 FIV classes against 18 different feline A3Z3 proteins.

## RESULTS

### Complicated evolution of the *A3Z3* genes in felids.

**FIG 1:**
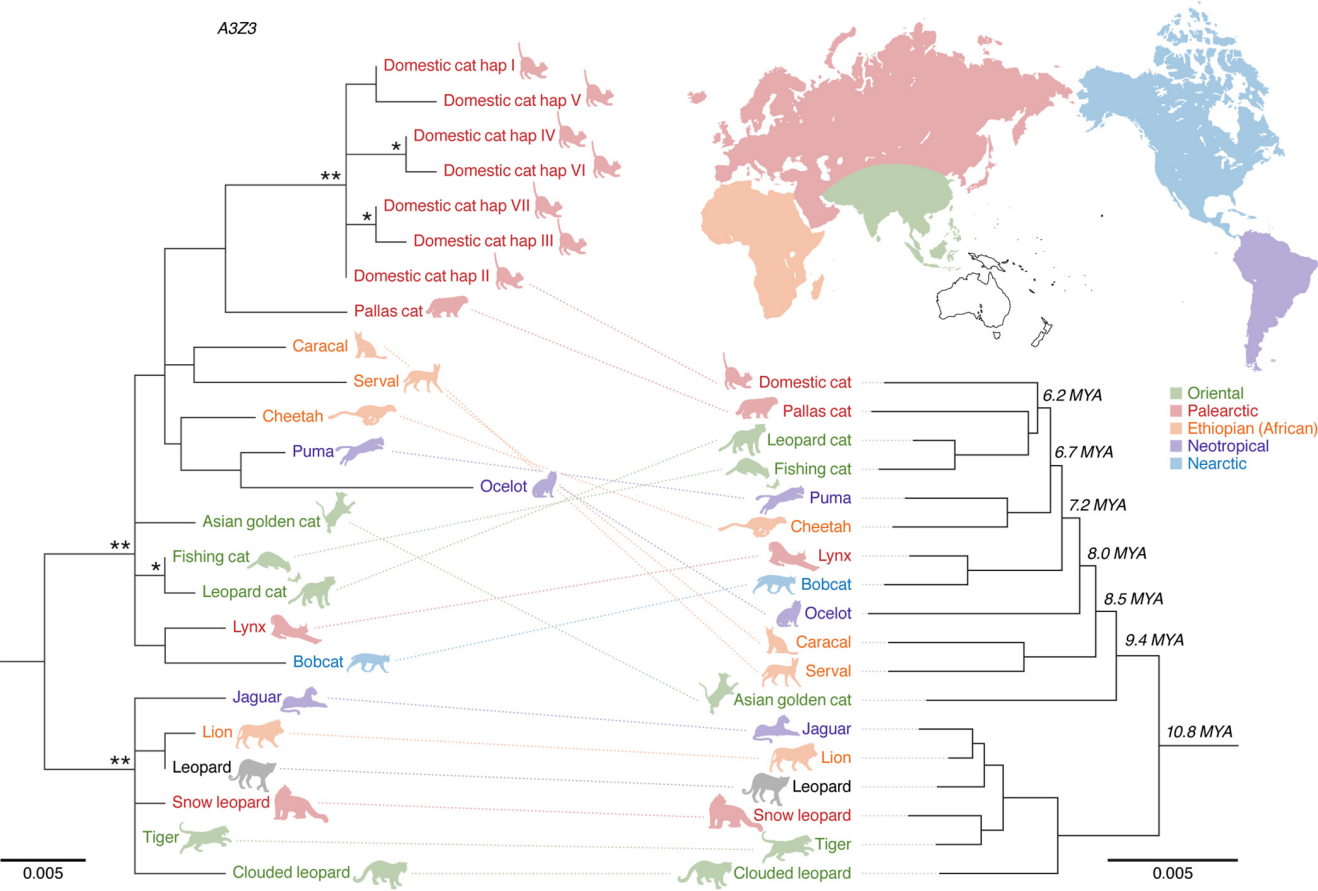
Molecular phylogenetic of feline *A3Z3* genes, phylogenetic relations of felid species, and the biogeographic distribution of felids. (Left) A maximum-likelihood tree of feline *A3Z3* genes of 18 felids. The *A3Z3* genes used are listed in Table 1. The phylogenetic tree was constructed as described in Materials and Methods. Bootstrap values are indicated as follows: *, >70%; **, >95%. Scale bar indicates 0.005 nucleotide substitutions per site. (Top right) The biogeographic distribution of felids. The data except for jaguar are from a previous study (15), and the distribution of jaguar is from the IUCN RED LIST (https://www.iucnredlist.org). Based on the previous report (15) and the IUCN RED LIST, the habitat of leopards is broad and spans African and Eurasian continents (Afro-Eurasia). (Bottom right) The phylogenetic relationship of felid species. The data are from a previous study (15). MYA, million years ago. Scale bar indicates 0.005 nucleotide substitutions per site. The colors of felid names and symbols are identical to those of their biogeographic distribution (top right).

The 18 feline species including focused in this study are listed in Table 1. In this study, we newly determined the *A3Z3* sequences of 11 felids: Pallas’s cat (*Otocolobus manul*), leopard cat (Prionailurus bengalensis), fishing cat (Prionailurus viverrinus), ocelot (Leopardus pardalis), caracal (Caracal caracal), serval (Leptailurus serval), Asian golden cat (Catopuma temminckii), jaguar (Panthera onca), leopard (Panthera pardus), snow leopard (Panthera uncia), and clouded leopard (Neofelis nebulosa) (Table 1). We generated the multiple-sequence alignments (MSAs) of these *A3Z3* genes, constructed the maximum likelihood (ML) phylogenetic tree, and compared the phylogenetic topology of feline *A3Z3* genes with that of felid species (15). As shown in Fig. 1, the *A3Z3* genes of the *Panthera* lineage (including jaguar, lion, leopard, snow leopard, tiger, and clouded leopard) corresponded well to the phylogenetic relationship of species classification. On the other hand, the *A3Z3* genes of non-*Panthera* lineages were incongruent with the species phylogeny (Fig. 1). Particularly, the phylogenetic positions of the *A3Z3* genes of lynx and bobcat are clearly different from those in the species phylogeny (Fig. 1). We then assessed the possibility of geographical convergence of feline *A3Z3* genes according to a previous study (15). However, there were no associations between the diversification of feline *A3Z3* genes with the biogeographical region of feline habitat (Fig. 1).

**TABLE 1:** Summary of the felids and their *A3Z3* genes in this study

Species name	Scientific name	Lineage^*a*^	Habitat^*a*^	Accession no.	Tissues used	Provider
Domestic cat	Felis catus	Domestic cat	Palearctic	EU011792 ^*b*^		
Pallas’s cat	*Otocolobus manul*	Leopard cat	Palearctic	LC597243 ^*c*^	Lung	Takayuki Miyazawa
Leopard cat	*Prionailurus bengalensis*	Leopard cat	Oriental	LC597240 ^*c*^	Blood	Inokashira Park Zoo, Tokyo, Japan
Fishing cat	*Prionailurus viverrinus*	Leopard cat	Oriental	LC597238 ^*c*^	Body hair	Osaka Municipal Tennoji Zoo, Osaka, Japan
Puma	*Puma concolor*	Puma	Neotropical	GU097659 ^*d*^		
Cheetah	Acinonyx jubatus	Puma	African	LC376039 ^*e*^		
Lynx	Lynx lynx	*Lynx*	Palearctic	GU097661 ^*d*^		
Bobcat	*Lynx rufus*	*Lynx*	Nearctic	LC376040 ^*e*^		
Ocelot	*Leopardus pardalis*	Ocelot	Neotropical	LC597242 ^*c*^	Muscle	Kanazawa Zoo, Kanagawa, Japan
Caracal	*Caracal caracal*	Caracal	African	LC597236 ^*c*^	Body hair	A pet shop, Shizuoka, Japan
Serval	*Leptailurus serval*	Caracal	African	LC597244 ^*c*^	Body hair	Hamura Zoo, Tokyo, Japan
Asian golden cat	*Catopuma temminckii*	Bay cat	Oriental	LC597235 ^*c*^	Muscle	Osaka Municipal Tennoji Zoo, Osaka, Japan
Lion	*Panthera leo*	*Panthera*	African	GU097662 ^*d*^		
Jaguar	*Panthera onca*	*Panthera*	Nearctic	LC597239 ^*c*^	Body hair	Osaka Municipal Tennoji Zoo, Osaka, Japan
Leopard	*Panthera pardus*	*Panthera*	African, Palearctic, and Oriental	LC597241 ^*c*^	Muscle	Osaka Municipal Tennoji Zoo, Osaka, Japan
Tiger	*Panthera tigris*	*Panthera*	Oriental	GU097663 ^*d*^		
Snow leopard	*Panthera uncia*	*Panthera*	Palearctic	LC597245 ^*c*^	Liver	Asahiyama Zoo, Hokkaido, Japan
Clouded leopard	*Neofelis nebulosa*	*Panthera*	Oriental	LC597237 ^*c*^	Muscle	Kanazawa Zoo, Kanagawa, Japan

a Reference 15.

b Reference 58.

c This study.

d Reference 23.

e Reference 36.

**FIG 2:**
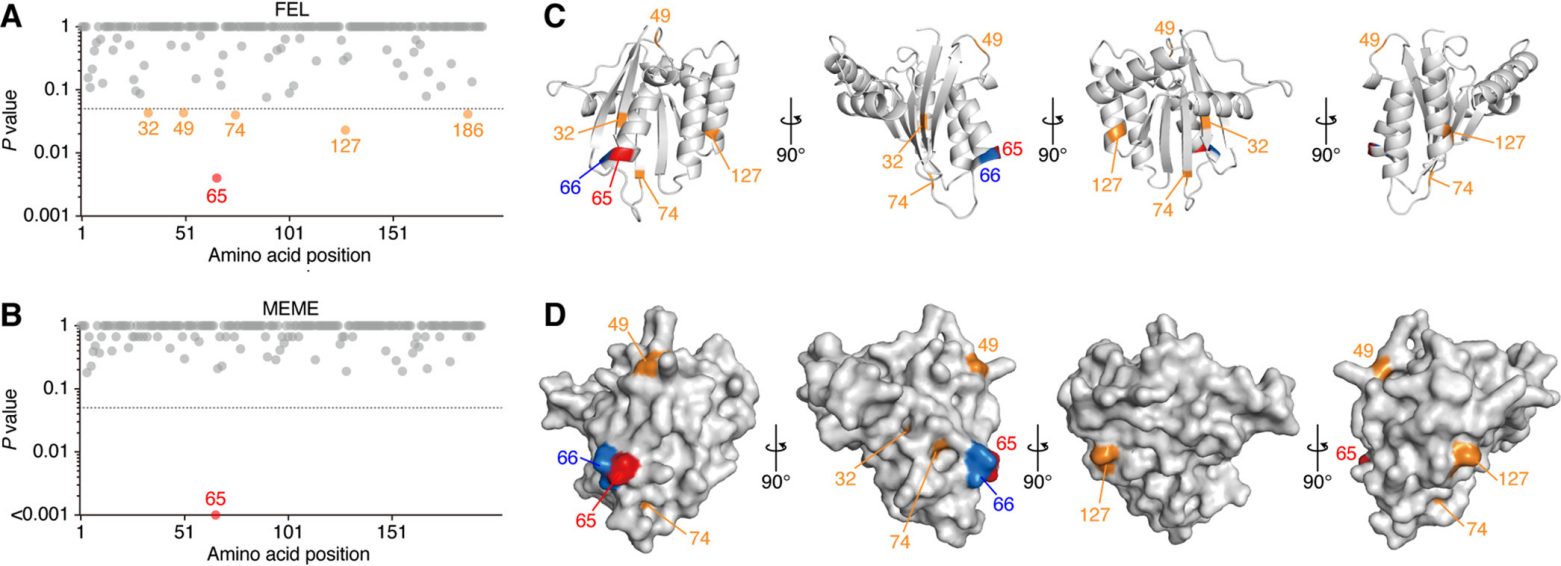
Molecular evolution of feline A3Z3 genes and the position of the residues under diversifying selection. (A and B) Codon sites under diversifying selection in feline *A3Z3* genes. The *P* value for each codon site is plotted. The site with indel mutation was omitted from the result. The results by FEL (37) (A) and MEME (38) (B) are shown. FEL and MEME are specialized for detecting the selections that occurred in the entire phylogenetic tree and the specific clades, respectively. (C and D) Structure modeling of domestic cat A3Z3 (hap I). The data of the structure homology model are from our previous study (22). Cartoon (C) and surface (D) models of the structure of domestic cat A3Z3 hap I are shown. In panels C and D, the residue under diversifying selection estimated by both FEL and MEME (residue 65) and those by FEL (residues 32, 49, 74, and 127) are represented in red and orange, respectively. Residue 66 is labeled in blue. Note that the residue 186 is not plotted, because this residue is located outside this homology model.

Next, we performed evolutionary analysis on feline *A3Z3* genes. The MSAs of feline A3Z3 proteins showed that an amino acid, asparagine, is inserted at position 24 of all A3Z3 proteins belonging to the *Panthera* lineage. In the non-*Panthera* lineages, only cheetah possessed an additional amino acid, tyrosine, at position 24. The ratio of nonsynonymous to synonymous evolutionary changes (*dN/dS* ratio) analyses by two independent methods, fixed-effects likelihood (FEL) (37) and mixed-effects model of evolution (MEME) (38), revealed that the amino acid at position 65 of feline A3Z3 is under strong diversifying selection (Fig. 2A and B; note that the amino acid position of feline A3Z3 protein mentioned in this study is based on that of domestic cat A3Z3 protein). Because the *dN/dS* analysis is not applicable for singleton indel sites, a one-amino-acid insertion at position 24 in the *Panthera* lineage and cheetah is removed from these analyses). These results correspond well to our previous findings showing that the amino acid at position 65 of domestic cat A3Z3 is under strong diversifying selection (22). Additionally, the analysis by FEL showed that the residues at positions 32, 49, 65, 74, 127, and 186 are under diversifying selection (Fig. 2A). By plotting these residues on the protein homology model of domestic cat A3Z3 protein (22), we found that residue 65 and the four additional residues, positioned at 32, 49, 74, and 127, are located on the protein surface (Fig. 2C and D).

### Different anti-FIV capacities of feline A3Z3 proteins.

**FIG 3:**
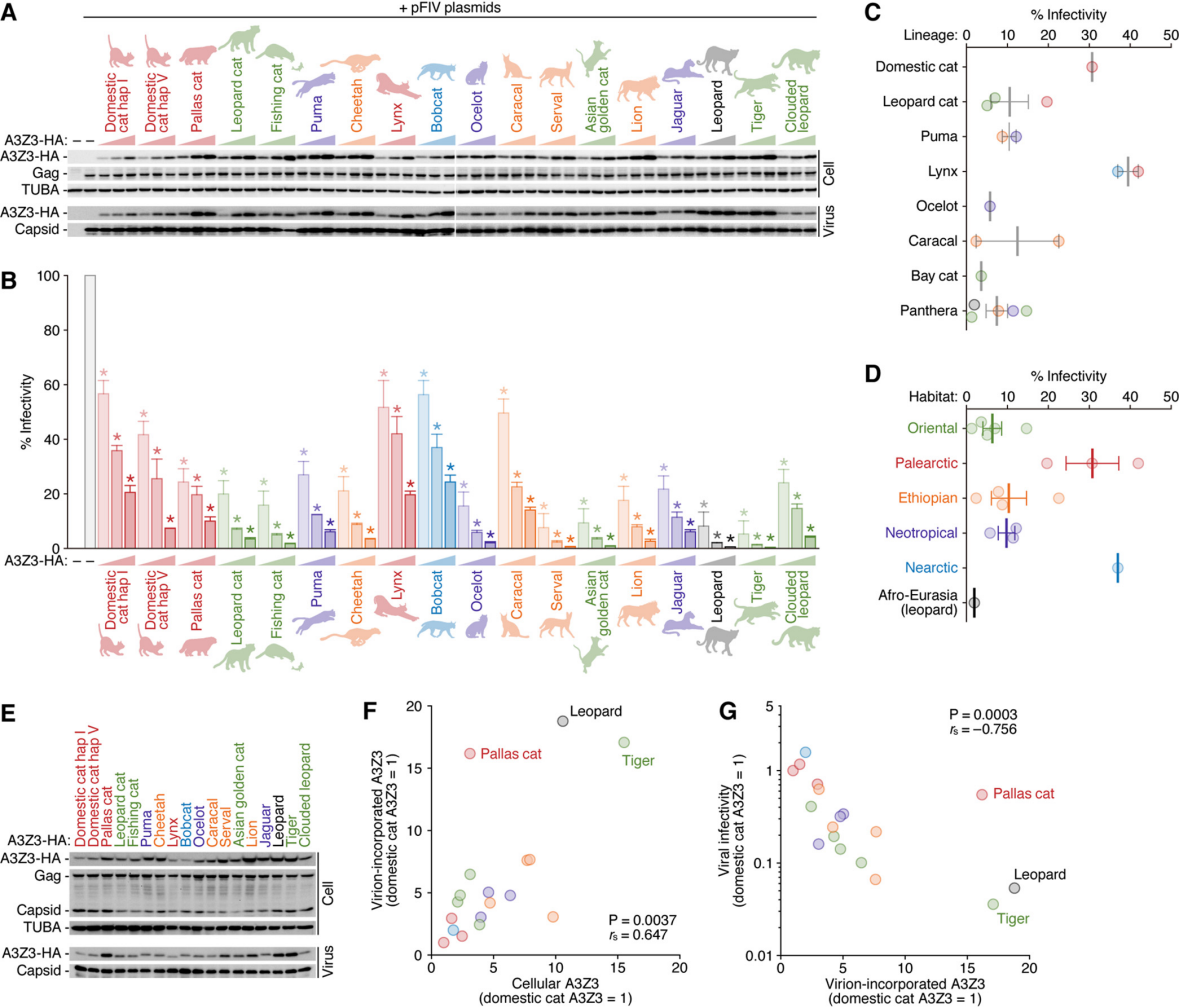
Antiviral activities of 18 feline A3Z3 proteins. (A and B) HA-tagged expression plasmids for feline A3Z3 (0, 100, 200, and 400 ng) and the three plasmids to produce the *vif*-deficient FIV-based reporter virus (FIV plasmids: pFP93 [200 ng], pTiger-luc [150 ng], and pMD.G [50 ng]) were cotransfected into HEK293T cells. (A) Western blotting. Representative results of three independent experiments are shown. Gag, FIV Gag precursor. (B) FIV reporter assay. FIV infectivity is shown as the percentage of the value without A3Z3. *, *P* < 0.05 by Student’s *t* test versus the value without A3Z3. The assays were independently performed in triplicates, and the averages with SEMs are shown. (C and D) Associations of the antiviral activity of feline A3Z3 with the lineage and the habitat of felids. The data of the viral infectivity at 200 ng of the HA-tagged feline A3Z3 expression plasmid shown in panel B (the middle column of each felid) were resorted based on the lineage (C) and the habitat (D) of each felid. Each classification is based on the information summarized in Table 1. For domestic cat, the average value of hap I and hap V is used. Each dot represents the value from each species, and the vertical bars indicate the averages with SEMs. (E) Western blotting. The samples at 200 ng of the HA-tagged feline A3Z3 expression plasmid shown in panel A (the middle column of each felid) were reblotted for the comparison. Representative results of at least three independent experiments are shown. Gag, FIV Gag precursor. (F and G) Correlations between the levels of feline A3Z3 proteins and viral infectivity. (F) Correlation between the levels of A3Z3 proteins in the cells (*x* axis) and those in the virions (*y* axis). The levels of cellular A3Z3 and virion-incorporated A3Z3 were normalized to the levels of TUBA and viral capsid, respectively. (G) Correlation between the levels of A3Z3 proteins in the virions (*x* axis) and viral infectivities (*y* axis). In panels F and G, the values of domestic cat hap I are set to 1. Each dot represents the value from each species. Each parameter was extracted from the data at 200 ng of the HA-tagged feline A3Z3 expression plasmid shown in panels A and B (the middle column of each felid). The parameters were extracted from the three independent experiments, and the average values are plotted.

We next investigated the anti-FIV ability of these 18 different feline A3Z3 proteins. Because the amino acid sequence of the snow leopard A3Z3 protein is identical to that of the tiger A3Z3 protein, we excluded the snow leopard A3Z3 protein from the cell culture experiments. Instead, we included two haplotypes of domestic cat A3Z3, hap I and hap V (22). The hemagglutinin (HA)-tagged expression plasmids for 18 feline A3Z3 proteins were constructed and were respectively cotransfected with *vif*-deficient FIV-based reporter plasmids at three different doses as previously described (22, 24, 36). As shown in Fig. 3A, all feline A3Z3 proteins were expressed and incorporated into the released virions in a dose-dependent manner. Feline A3Z3 expression did not affect the expression level of FIV Gag precursor in virus-producing cells or the amount of released viral particles (Fig. 3A). Furthermore, the virion-incorporated feline A3Z3 proteins suppressed viral infectivity in a dose-dependent manner (Fig. 3B). Because the antiviral activities of feline A3Z3 proteins were different from each species (Fig. 3B), we assessed the association of the antiviral activity of feline A3Z3 proteins with the lineage and the habitat of felids. As shown in Fig. 3C, the five A3Z3 proteins of the *Panthera* lineage (lion, jaguar, leopard, tiger, and clouded leopard) and the two A3Z3 proteins of the *Puma* lineage (puma and cheetah) tended to exhibit relatively higher anti-FIV activity. On the other hand, the antiviral activities of the two A3Z3 proteins of the *Lynx* lineage (lynx and bobcat) were relatively low (Fig. 3C). Correlation analysis with the felid habitat showed that the A3Z3 proteins of the felids living in the Oriental (leopard cat, fishing cat, Asian golden cat, tiger, and clouded leopard), Ethiopian (cheetah, caracal, serval, and lion) and Neotropical (puma and ocelot) regions tended to exhibit relatively higher anti-FIV activity (Fig. 3D).

In addition to the differences in the antiviral activities of each feline A3Z3 protein (Fig. 3B), the expression levels of feline A3Z3 proteins in the cells and the efficacy for virion incorporation of these proteins are different among species (Fig. 3E). As shown in Fig. 3F, there was a significant correlation between the levels of feline A3Z3 proteins in the cells and the virions (*P* = 0.0037, *r *=* *0.647 by Spearman’s rank correlation coefficient). Particularly, the A3Z3 proteins of some felids of the *Panthera* lineage such as leopard and tiger were expressed relatively highly and incorporated efficiently into the released virions (Fig. 3F). In contrast, Pallas’s cat A3Z3 protein was incorporated into the released virions relatively efficiently irrespective of its relatively low level of expression in cells (Fig. 3F). When we assessed the correlation between the level of virion-incorporated A3Z3 protein and the infectivity of the released virions, there was a significant and negative correlation between the level of virion-incorporated A3Z3 protein and viral infectivity (Fig. 3G). As expected, the A3Z3 proteins of leopard and tiger, which were incorporated efficiently into the released virions, exhibited higher antiviral activity (Fig. 3G). In contrast, Pallas’s cat A3Z3 showed relatively low antiviral activity per the amount of A3Z3 protein in the virion (Fig. 3G).

### Feline A3Z3 antagonism by a variety of FIV Vif proteins.

**FIG 4:**
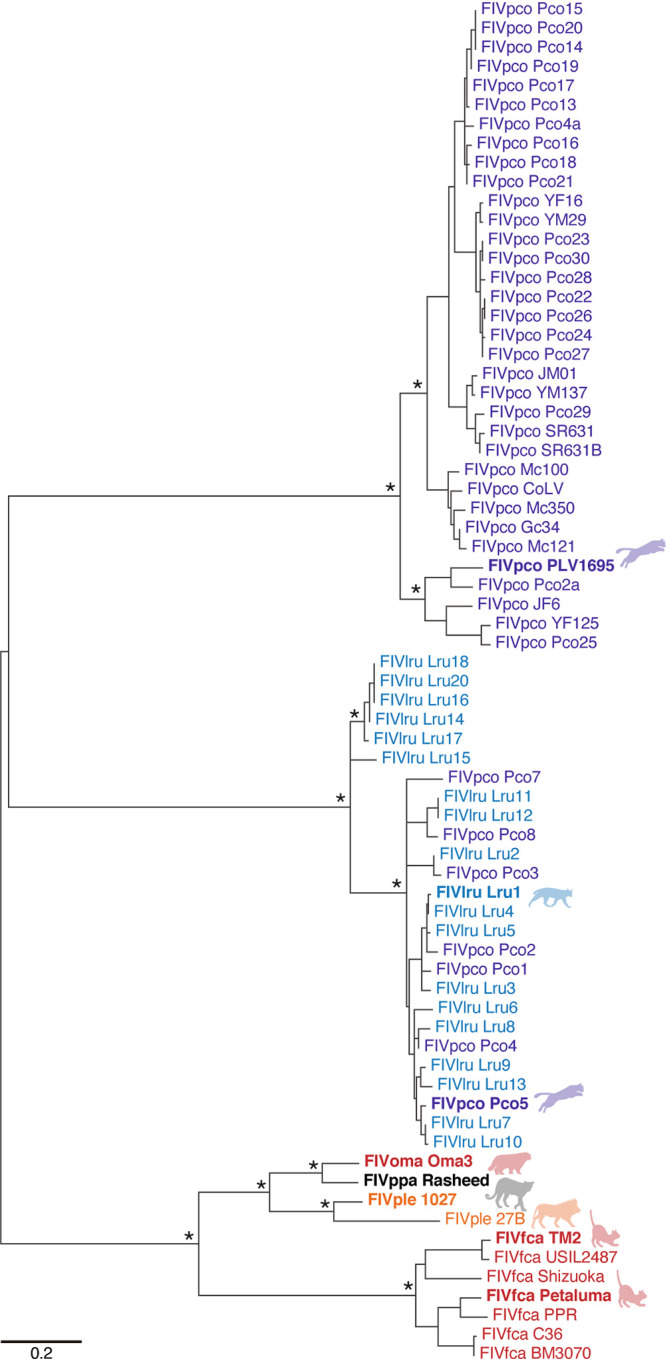
Molecular phylogenetic of FIV Vif. A maximum likelihood tree of 70 FIV Vif proteins from 6 different host felids. The information of FIV Vif is listed in Table 2. The phylogenetic tree was constructed as described in Materials and Methods. Bootstrap values are indicated as follows: *, >95%. Scale bar indicates 0.2 amino acid substitutions per site. The FIV Vif proteins used for the cell culture experiments are indicated in bold.

To reveal the functional relationship between feline A3Z3 and the Vif proteins of a variety of FIV classes, we constructed a phylogenetic tree of the Vif proteins of the 70 different strains of FIV listed in Table 2. In addition to the *vif* genes from the 5 FIV lineages in 5 felids, we newly determined an FIV *vif* sequence from a leopard (FIVppa, strain Rasheed). This is the first FIV *vif* sequence obtained from leopard, and the phylogenetic tree showed that FIVppa Vif is similar to FIVoma Vif (Fig. 4). This is consistent with a previous study (39) showing that the sequence of the reverse transcriptase (RT)-pol region of FIVoma is close to that of FIVppa.

**TABLE 2:** Accession numbers of FIV *vif* sequences used in this study

Viral class	Strain	Accession no.
FIVfca	TM2	M59418
FIVfca	USIL2487	U11820
FIVfca	Shizuoka	LC179609
FIVfca	Petaluma	M25381
FIVfca	PPR	M36968
FIVfca	C36	AY600517
FIVfca	BM3070	AF474246
FIVoma	Oma3	AY713445
FIVlru	Lru1	KF906143
FIVlru	Lru2	KF906144
FIVlru	Lru3	KF906145
FIVlru	Lru4	KF906146
FIVlru	Lru5	KF906147
FIVlru	Lru6	KF906148
FIVlru	Lru7	KF906149
FIVlru	Lru8	KF906150
FIVlru	Lru9	KF906151
FIVlru	Lru10	KF906152
FIVlru	Lru11	KF906153
FIVlru	Lru12	KF906154
FIVlru	Lru13	KF906155
FIVlru	Lru14	KF906156
FIVlru	Lru15	KF906157
FIVlru	Lru16	KF906158
FIVlru	Lru17	KF906159
FIVlru	Lru18	KF906160
FIVlru	Lru20	KF906162
FIVpco	CoLV	EF455615
FIVpco	PLV1695	DQ192583
FIVpco	Gc34	EF455603
FIVpco	JF6	EF455610
FIVpco	JM01	EF455609
FIVpco	Mc100	EF455605
FIVpco	Mc121	EF455606
FIVpco	Mc350	EF455604
FIVpco	SR631	EF455613
FIVpco	SR631B	EF455614
FIVpco	YF16	EF455608
FIVpco	YF125	EF455612
FIVpco	YM29	EF455607
FIVpco	YM137	EF455611
FIVpco	Pco1	KF906163
FIVpco	Pco2	KF906164
FIVpco	Pco2a	KF906185
FIVpco	Pco3	KF906165
FIVpco	Pco4	KF906166
FIVpco	Pco4a	KF906181
FIVpco	Pco5	KF906167
FIVpco	Pco7	KF906169
FIVpco	Pco8	KF906170
FIVpco	Pco13	KF906180
FIVpco	Pco14	KF906182
FIVpco	Pco15	KF906183
FIVpco	Pco16	KF906193
FIVpco	Pco17	KF906175
FIVpco	Pco18	KF906176
FIVpco	Pco19	KF906177
FIVpco	Pco20	KF906178
FIVpco	Pco21	KF906179
FIVpco	Pco22	KF906188
FIVpco	Pco23	KF906189
FIVpco	Pco24	KF906190
FIVpco	Pco25	KF906191
FIVpco	Pco26	KF906192
FIVpco	Pco27	KF906194
FIVpco	Pco28	KF906184
FIVpco	Pco29	KF906186
FIVpco	Pco30	KF906187
FIVple	1027	EU117992
FIVppa	Rasheed	LC599586 ^*a*^

a This study.

The Vif sequences of FIVfca, FIVple, and FIVlru formed respective clusters according to their host species (i.e., domestic cats, lions, and bobcats) (Fig. 4). Consistent with previous reports (28, 34–36), FIVpco Vif sequences were classified into two distinct clusters, and a cluster mingled with FIVlru Vif (Fig. 4). This observation suggests the intergenus circulation of a subclass of FIVpco between pumas and bobcats in the wild (28, 34, 36).

**FIG 5:**
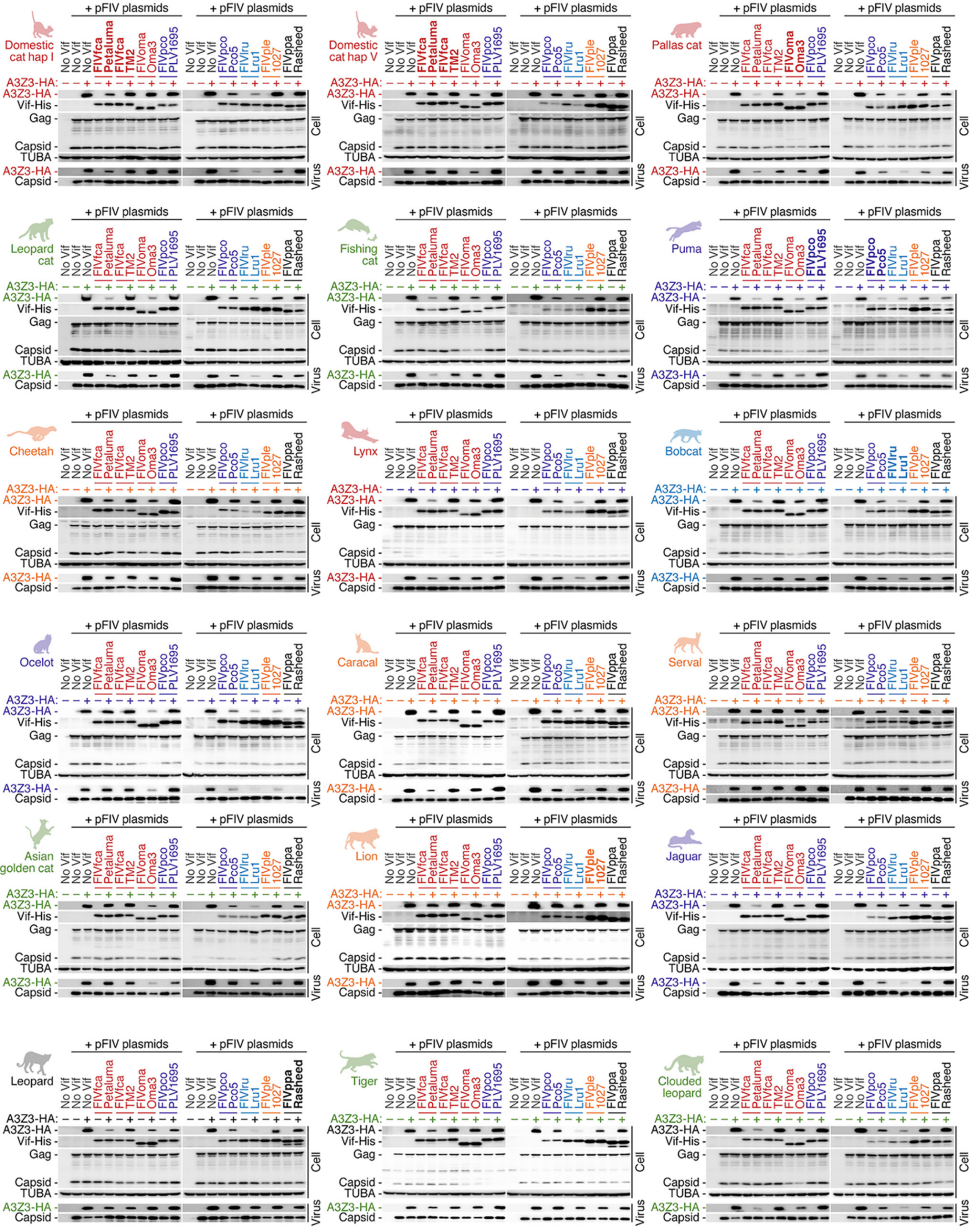
Evaluation of the ability of eight FIV Vif proteins to degrade 18 kinds of feline A3Z3 proteins. HA-tagged expression plasmids for feline A3Z3 (200 ng) and the three plasmids to produce the *vif*-deficient FIV-based reporter virus (FIV plasmids: pFP93 [200 ng], pTiger-luc [150 ng] and pMD.G [50 ng]) were cotransfected with or without His-tagged FIV Vif expression plasmids (400 ng) into HEK293T cells. Representative results of three independent experiments are shown. The FIV Vif isolated from its host species is indicated in bold. Gag, FIV Gag precursor.

**FIG 6:**
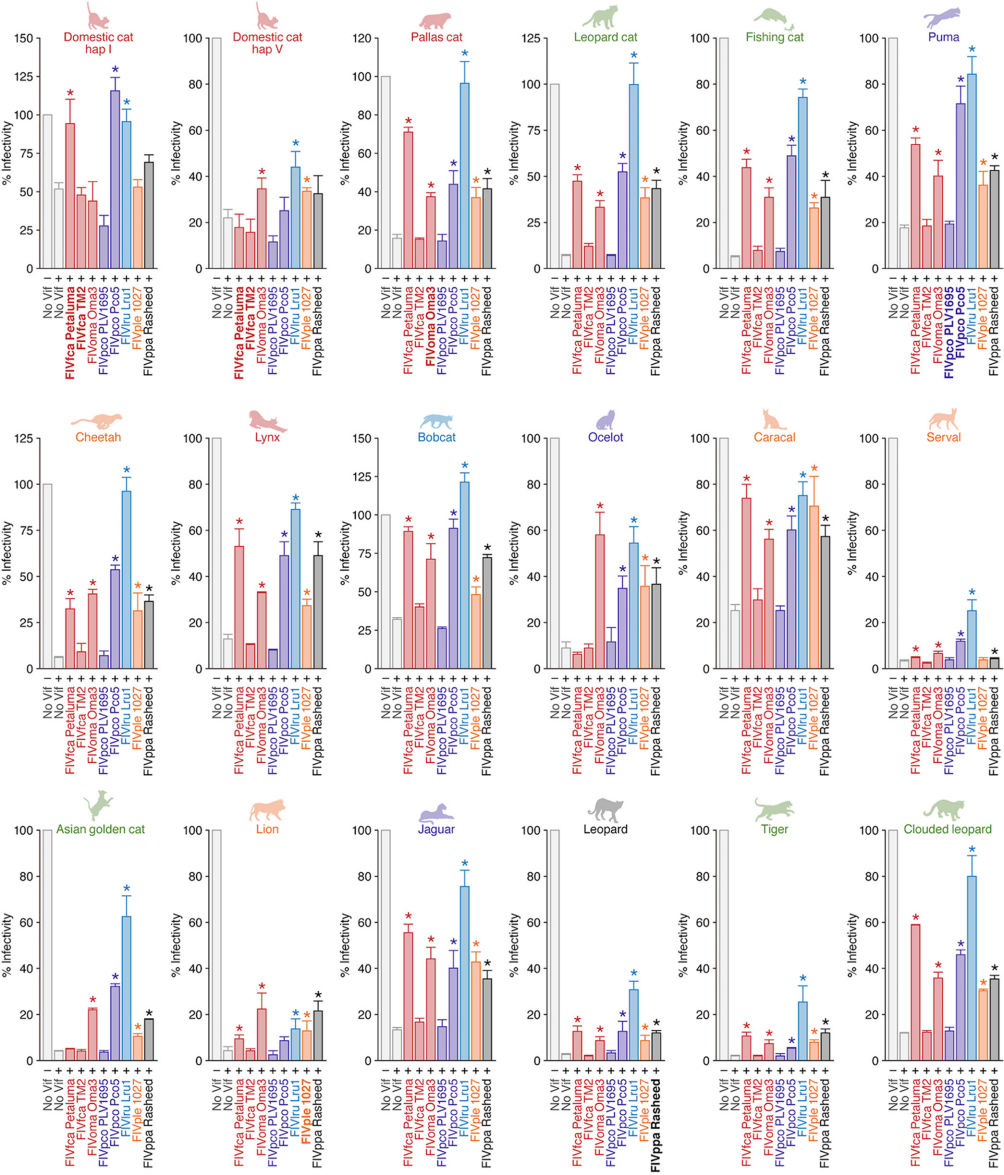
Evaluation of the counteracting ability of eight FIV Vif proteins against 18 kinds of feline A3Z3 proteins. The experimental setup is the same as that of Fig. 5. FIV infectivity is shown as the percentage of the value without each FIV Vif. “−” or “+” on the *x* axes represents the absence or presence of the HA-tagged expression plasmid for the feline A3Z3 indicated in each graph. *, *P* < 0.05 by Student’s *t* test versus “no Vif.” The assays were independently performed in triplicates, and the averages with SEMs are shown. The FIV Vif isolated from its host species is indicated in bold.

From these 70 FIV *vif* sequences, we picked up eight representative FIV Vif proteins from six FIV lineages: FIVfca strains Petaluma (subtype A) and TM2 (subtype B) (22, 24), FIVoma strain Oma3, FIVpco strains PLV1695 and Pco5 (36, 40), FIVlru strain Lru1 (36), FIVple strain 1027, and FIVppa strain Rasheed. We prepared His-tagged Vif expression plasmids and used them for cell culture experiments (Fig. 5 and 6). Western blotting showed that all eight FIV Vif proteins expressed efficiently (Fig. 5). Also, the expression of FIV Vif tested did not affect the expression level of Gag precursor in transfected cells or the amount of released viral particles (Fig. 5). However, two FIV Vif proteins, FIVfca TM2 and FIVpco PLV1695, were unable to degrade any feline A3Z3 proteins (Fig. 5). These results are reminiscent of the previous findings that FIVfca TM2 (24) and FIVpco PLV1695 (40) Vifs cannot degrade the APOBECs of their respective host felids (i.e., domestic cat and puma). FIV reporter assays showed that the A3Z3 proteins of some felids such as serval, lion, and leopard were relatively resistant to the counteraction by FIV Vif proteins (Fig. 6). However, the four FIV Vif proteins tested (i.e., except for FIVfca TM2 and FIVpco PLV1695) were able to counteract the antiviral activity of most feline A3Z3 proteins (Fig. 6).

### Resistance of the A3Z3 proteins of ocelot and Asian golden cat to FIVfca Petaluma Vif.

**FIG 7:**
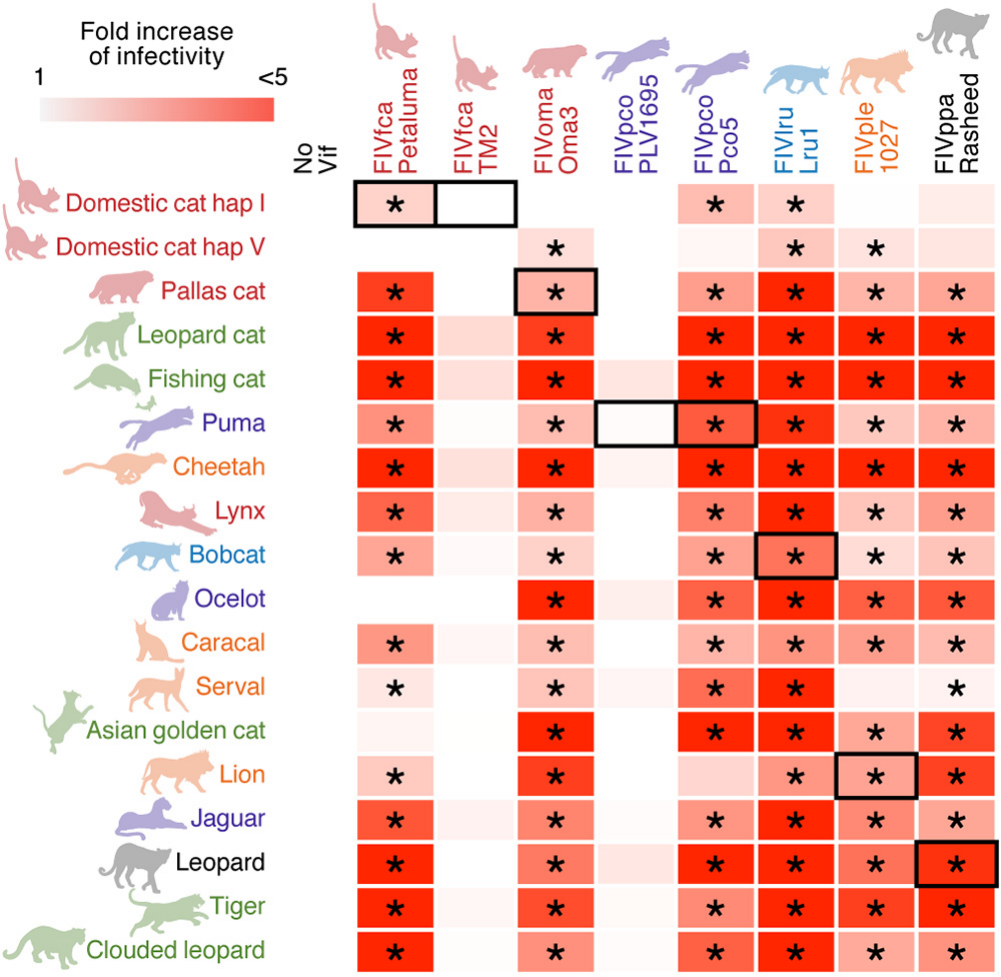
A heat map of antiviral activity of 18 feline A3Z3s and their counteraction by eight FIV Vifs. The data shown in Fig. 7 are summarized as a heat map. *, *P* < 0.05 by Student’s *t* test versus “no Vif.” The results from the interplay between FIV and its natural host are indicated by black outlines.

For the sake of simplicity, we summarized the data as a heat map (Fig. 7). This heat map showed that FIVlru Vif (strain Lru1) is able to counteract the antiviral activity of all feline A3Z3 proteins tested (Fig. 7). We also found that the Vif proteins of FIVpco Pco5, FIVple 1027, and FIVppa Rasheed can counteract most of the feline A3Z3 proteins tested (Fig. 7). Consistent with our previous study (22), domestic cat A3Z3 hap V protein was resistant to counteraction by FIVfca Petaluma Vif, while domestic cat A3Z3 hap I protein was sensitive (Fig. 5 and 7). Interestingly, we found that the A3Z3 proteins of ocelot and Asian golden cat were also resistant to FIVfca Petaluma Vif-mediated counteraction (Fig. 7).

**FIG 8:**
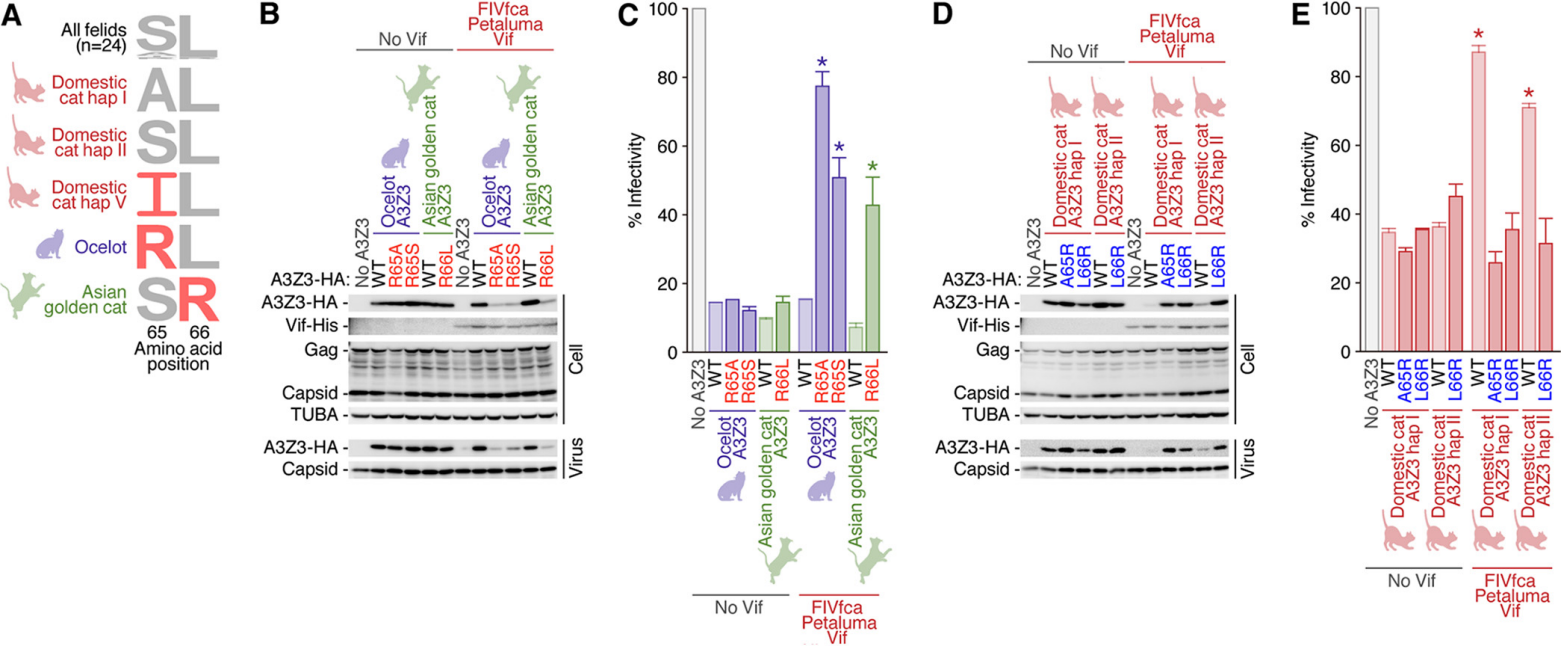
Determinants of feline A3Z3 sensitivity to FIVfca Petaluma Vif-mediated counteraction. (A) Logo plot. The amino acid residues at positions 65 and 66 of all feline A3Z3 proteins used in this study (*n* = 24), domestic cat hap I, II, and V, ocelot, and Asian golden cat, are shown. The residues that are responsible for the resistance to FIVfca Petaluma Vif-mediated counteraction are shown in red. (B to E) Western blotting and FIV reporter assay. Loss-of-function experiments using the three A3Z3 mutants (ocelot A3Z3 R65A and R65S and Asian golden cat A3Z3 R66L) (B and C) and gain-of-function experiments using the three domestic cat A3Z3 mutants (hap I A65R, hap I L66R, and hap II L66R) (D and E) were performed. (B and D) Western blotting. HA-tagged expression plasmids for feline A3Z3 (200 ng) and the three plasmids to produce the *vif*-deficient FIV-based reporter virus (FIV plasmids: pFP93 [200 ng], pTiger-luc [150 ng], and pMD.G [50 ng]) were cotransfected with or without His-tagged FIVfca Petaluma Vif expression plasmid (400 ng) into HEK293T cells. Representative results of three independent experiments are shown. (C and E) FIV reporter assay. FIV infectivity is shown as the percentage of the value of “no A3Z3.” *, *P* < 0.05 by Student’s *t* test versus without Vif. The assays were independently performed in triplicates, and the averages with SEMs are shown.

It has been demonstrated that only one amino acid at position 65 is different between domestic cat A3Z3 hap I (65A) and hap V (65I) proteins (22). This previous finding indicated that residue 65 of domestic cat A3Z3 protein determines the sensitivity to FIVfca Petaluma Vif. When we assessed the MSA of feline A3Z3 proteins, we found that only ocelot A3Z3 protein possesses arginine (R) at position 65 while only Asian golden cat A3Z3 protein possesses R at position 66 (Fig. 8A). To address the possibility that the residues at positions 65 to 66 of the A3Z3 proteins of ocelot and Asian golden cat determine their resistance to FIVfca Petaluma Vif-mediated degradation, we prepared expression plasmids for the three A3Z3 mutants: ocelot A3Z3 R65A and R65S and Asian golden cat A3Z3 R66L. As shown in Fig. 8B, these three A3Z3 protein mutants were degraded by FIVfca Petaluma Vif, although parental ocelot and Asian golden cat A3Z3 proteins were not. Furthermore, the FIV reporter assay revealed that the anti-FIV ability of these three A3Z3 mutants was canceled by FIVfca Petaluma Vif (Fig. 8C). To further test this issue, we prepared expression plasmids for the three domestic cat A3Z3 mutants: hap I A65R, hap I L66R, and hap II L66R. As expected, these three mutated A3Z3 proteins of domestic cats were resistant to FIVfca Petaluma Vif-mediated degradation (Fig. 8D). These A3Z3 proteins were incorporated into the released virions even in the presence of FIVfca Petaluma Vif (Fig. 8D) and significantly suppressed viral infectivity (Fig. 8E). Taken together, these results suggest that the residues at positions 65 and 66 of feline A3Z3 proteins determine sensitivity to FIVfca Petaluma Vif.

## DISCUSSION

In this study, we determined the sequences of 11 novel feline *A3Z3*s and demonstrated that all 18 different feline A3Z3 proteins tested exhibit anti-FIV activity (Fig. 1 and 3). Because the A3Z3 proteins of the felids that did not show FIV infections in the past (e.g., leopard cat, fishing cat, serval, and caracal) (27) also significantly suppressed the infectivity of *vif*-deleted FIV (Fig. 3), our data suggest that the anti-FIV activity of A3Z3 proteins is broadly maintained in all felids. Additionally, we used the eight FIV Vif expression plasmids from six different classes (FIVfca, FIVoma, FIVpco, FIVlru, FIVple, and FIVppa) and, particularly, newly determined one sequence (FIVppa) (Fig. 4). Using these materials, we performed 144 patterns of the round robin test using 18 feline A3Z3 proteins and 8 FIV Vif proteins (Fig. 5 and 6) and derived a matrix showing the interplay between feline A3Z3 and FIV Vif (Fig. 7). To our knowledge, this is the first study comprehensively investigating the functional interplay between feline A3Z3 proteins and FIV Vif proteins. Based on these analyses, particularly, on the interplay between the A3Z3 proteins of domestic cat, ocelot, and Asian golden cat and FIVfca Petaluma Vif, we revealed that residues 65 and 66 of the A3Z3 proteins of multiple felids are responsible for the counteraction triggered by FIVfca Petaluma Vif (Fig. 8).

Similar to the observations on the *A3* genes in primates (11) and bovids (13), the phylogenetic relationship of most of the feline *A3Z3* genes was incongruent with the species phylogeny of felids (Fig. 1). However, the six *A3Z3* genes of the *Panthera* lineage (jaguar, lion, leopard, snow leopard, tiger, and clouded leopard) form an independent cluster, and the phylogeny of the *A3Z3* genes of the *Panthera* lineage was identical to their species phylogeny (Fig. 1). It was also intriguing that the amino acid sequences of tiger A3Z3 and snow leopard A3Z3 proteins were identical. Although intraspecies polymorphisms in *A3* genes have been reported in human *A3H* (19, 41–44), African green monkey *A3G* (11, 12), and domestic cat *A3Z3* (16, 22, 36), this is the first report showing that the amino acid sequences of the A3Z3 proteins from different species are identical. Moreover, the anti-FIV activity of the A3Z3 proteins of the *Panthera* lineage tended to be commonly higher than those of non-*Panthera* lineages (Fig. 2C). These findings suggest that the antiviral activity of the A3Z3 proteins of the *Panthera* lineage has been maintained and sophisticated in a long evolutionary history of approximately 10.8 million years. On the other hand, the phylogenetic relationship of the *A3Z3* genes of the *Lynx* lineage (lynx and bobcat) was clearly inconsistent with the species phylogeny (Fig. 1), and the antiviral activity of these A3Z3 proteins was commonly lower than those of the other feline A3Z3 proteins (Fig. 3C). Regarding this issue, a previous report showed that the common ancestor of the *Lynx* lineage diversified from the other felid species approximately 7.2 MYA, and this ancestor lived in North America until the diversification of lynx and bobcat as independent species approximately 3.2 MYA (15). A possible explanation for the unique properties of the A3Z3 proteins of the *Lynx* lineage is that the antiviral capacity of the A3Z3 proteins of the *Lynx* lineage has degenerated during this period, because the common ancestor of the *Lynx* lineage was free from pathogenic FIV infection. As another feature of the felids belonging to the *Lynx* lineage, bobcats and lynxes are the only felids with a short “bobbed” tail (45). Although the reason why their tails were shortened during the evolution and its association with the unique feature of the *A3Z3* genes in terms of the phylogeny (Fig. 1) and the antiviral ability (Fig. 3C) remain unclear, the feature of the *A3Z3* genes may be one of the consequences of the unique evolution of the *Lynx* lineage.

Here, we determined the novel sequence of FIVppa Vif and revealed that FIVppa Vif is phylogenetically similar to FIVoma Vif (Fig. 4). Our finding is consistent with a previous report showing that the RT-pol region of FIVppa is similar to that of FIVoma (39). These observations suggest that FIVppa and FIVoma share an origin. To assume the direction of the cross-species transmission, there might be two possibilities: first, Pallas’s cats, the host of FIVoma, only live in Asia, while the habitat of leopards, the host of FIVppa, extends across both Africa and Eurasia. Therefore, there would be relatively more frequent opportunities for leopards to be infected with a variety of pathogens, including FIVs, and one possibility is that FIVoma has emerged by the cross-species transmission of FIVppa from leopards to Pallas’s cats. Another possibility may be explained by the prey-predator relationship. This relationship partly explains the direction of cross-species transmission of SIVs from Old World monkeys to chimpanzees in the wild: because small Old World monkeys are the prey of chimpanzees in the wild, chimpanzees are frequently exposed to various SIVs that infect their prey species such as Old World monkeys (46, 47). Similarly, because Pallas’s cats are relatively small and can be the prey of leopards in the wild, leopards can be frequently exposed to FIVoma that infect Pallas’s cat, and this prey-predator relationship may have led to the emergence of FIVppa in leopards. In either case, the Vif proteins of both FIVoma and FIVppa are able to counteract the antiviral A3Z3 proteins of Pallas’s cats and leopards (Fig. 5 and 7). Therefore, our findings suggest that the A3Z3 proteins of Pallas’s cat and leopard cannot be the “species barrier” that hampers the cross-species transmission of FIVs.

Molecular evolutionary analyses revealed that at least six amino acid residues are under diversifying selection (Fig. 2). Particularly, residue 65 of domestic cat A3Z3 (identical to residue 66 of the MSA in this study) is the site that was determined as a strongly selected site in polymorphic domestic cat A3Z3 proteins and is responsible for resistance to degradation by FIVfca Petaluma Vif (22). In addition to residue 65, the other sites that are under diversifying selection by FEL (Fig. 2A) were exposed to the protein surface (Fig. 2D). Therefore, it would be possible to assume that an FIV Vif-like factor(s) has been the selective pressure of feline A3Z3 during the evolution of felids.

More intriguingly, we found that the A3Z3 proteins of ocelot and Asian golden cat are resistant to counteraction by FIVfca Petaluma Vif (Fig. 7) and demonstrated that the resistance of these A3Z3 proteins is determined by the amino acid residues at positions 65 and 66 (Fig. 8). Together with our previous findings (22), these results suggest that these two naturally occurring feline A3Z3 proteins confer resistance to infection with FIVfca. Because the lineage and biogeographical distribution of these three felids, domestic cat, ocelot, and Asian golden cat, are different (Fig. 1), the ability of A3Z3 to be resistant to FIVfca Petaluma Vif has been acquired independently and is a convergent evolution. Furthermore, Troyer et al. isolated an FIV from ocelot (27). Although its *vif* sequence was not determined, the phylogeny of the FIV pol-RT sequence showed that the FIV from ocelot is phylogenetically similar to FIVfca (27). These observations suggest that the FIVs from domestic cats and ocelots share their origin. Although FIV sequences have not been detected in Asian golden cats so far (27), future investigations on the interplay between the A3Z3 protein of ocelots and Asian golden cats and the Vif proteins of the FIV from these two felids will further reveal the interplay between felids and FIV with higher resolution.

Here, we performed a comprehensive investigation using expression plasmids for a variety of feline A3Z3 proteins and FIV Vif proteins and revealed new aspects on the roles of feline A3Z3 and FIV Vif on the cross-species transmission of lentiviruses. However, some issues could not to be elucidated due to technical limitations. For example, we found that the A3Z3 proteins of the *Panthera* lineage commonly exhibit relatively higher anti-FIV activity (Fig. 3C). Additionally, Pallas’s cat A3Z3 protein was efficiently incorporated into released virions but exhibited relatively lower antiviral activity (Fig. 3F and G). Moreover, some feline A3Z3 proteins exhibited resistance to counteraction by certain FIV Vif proteins (e.g., FIVple Vif versus domestic cat hap I A3Z3 and serval A3Z3, and FIVppa Vif versus domestic cat A3Z3) (Fig. 8). Although we revealed the residues of feline A3Z3 proteins that conferred resistance to FIVfca Petaluma Vif (Fig. 8), the MSA of feline A3Z3 proteins did not reveal which residues were responsible. It is possible that multiple amino acid residues of feline A3Z3 proteins contribute to its antiviral activity and resistance to FIV Vif. Furthermore, the amino acid residues on FIV Vif proteins that determine ability to counteract feline A3Z3 proteins were not addressed because of their higher diversity. Although we previously revealed that the residues at positions 167, 239, 242, and 243 of FIVfca subtype B Vif are responsible for losing the counteracting activity against domestic cat A3 proteins (24), the residue(s) that determines the loss of FIVpco PLV1695 Vif activity against feline A3 proteins remains veiled. To fully elucidate the complicated interplay between feline A3Z3 proteins and FIV Vif proteins, more detailed investigations will be needed. In addition to A3Z3, other intrinsic restriction factors such as tetherin (48, 49) and SAMHD1 (50, 51) in felids can be a barrier(s) against cross-species transmission of FIVs, while FIVs may possess viral counterparts to overcome these hurdles. Further investigations will be needed to unveil the interplay between FIV and felids.

In summary, here we performed a comprehensive investigation of the interplay between antiviral A3 proteins and lentiviruses in felids. As previously reported, the evolution of mammalian *A3* genes is complicated and is driven by certain selective pressures, including retroviruses (52–54). Our findings may provide an insight into both the scenarios of cross-species transmission of FIVs in felids and the evolutionary interactions between mammals and lentiviruses.

## MATERIALS AND METHODS

### Ethics statement.

To determine the 11 feline A3Z3 sequences, blood, body hair, or cryopreserved tissues from lung, liver, or muscle of Pallas’s cat (*Otocolobus manul*), leopard cat (*Prionailurus bengalensis*), fishing cat (*Prionailurus viverrinus*), ocelot (*Leopardus pardalis*), caracal (*Caracal caracal*), serval (*Leptailurus serval*), Asian golden cat (*Catopuma temminckii*), jaguar (*Panthera onca*), leopard (*Panthera pardus*), snow leopard (*Panthera uncia*), and clouded leopard (*Neofelis nebulosa*) were kindly provided by Takayuki Miyazawa (Kyoto University, Japan) or the following facilities: Asahiyama Zoo, Hokkaido, Japan; Kanazawa Zoo, Kanagawa, Japan; Hamura Zoo, Tokyo, Japan; Inokashira Park Zoo, Tokyo, Japan; Osaka Municipal Tennoji Zoo, Osaka, Japan; a pet shop in Shizuoka prefecture, Japan (listed in Table 1). Sampling in Japan was performed in accordance with the guidelines of Tokyo University of Agriculture, Japan. All experimental protocols in Japan were approved by the Animal Experiment Ethics Committee at the Tokyo University of Agriculture (approval number 300001).

FIVppa was isolated from a sample of leopard (*Panthera pardus*) blood submitted to the University of Glasgow for FIV diagnosis from the Breeding Centre for Endangered Arabian Wildlife in Sharjah, United Arab Emirates. Ethical approval for the use of residual feline blood submitted for routine diagnostic testing was granted by the ethics committee of the University of Glasgow School of Veterinary Medicine, UK.

### Sequencing PCR of feline *A3Z3* genes.

Sequencing PCR of feline *A3Z3* gene was performed as previously described (36). Briefly, genomic DNA was extracted from the samples described above using the DNA Extractor FM kit (Wako) or the DNeasy blood and tissue kit (Qiagen). PCR was performed using Prime-STAR GXL DNA polymerase (TaKaRa) and the following primers: feline *A3Z3* exon 2 forward (fwd), 5′-ACA AGA TGG GTG AGC CAA AG-3′; feline *A3Z3* exon 2 reverse (rev), 5′-CAG GGA TAT GAG GGG GTT CT-3′; feline *A3Z3* exon 3, 4 fwd, 5′-CCA GGT GAG TTC ACA GAG CA-3′; feline *A3Z3* exon 3, 4 rev, 5′-GGA CGG GTG TCT CAA GAA AA-3′; feline *A3Z3* exon 5 fwd, 5′-GCC TGT TTC CGA TTC TGT GT-3′; and feline *A3Z3* exon 5 rev, 5′-TTT ACG AAG GAA AGC CCT GA-3′. The obtained PCR products were purified by gel extraction using the QIAquick gel extraction kit (Qiagen). The nucleotide sequences were determined by a DNA sequencing service (Fasmac, Kanagawa, Japan), and the data were analyzed using Sequencher v5.1 software (Gene Codes Corporation).

To validate the species of the feline materials, the specimens’ *CYTB* sequences were analyzed by PCR and Sanger sequencing using the following primers as previously described (13, 55): forward (L14724), 5′-GAT ATG AAA AAC CAT CGT TG-3′, and reverse (H15149), 5′-CTC AGA ATG ATA TTT GTC CTC A-3′. We then confirmed the species of the specimens used.

### Molecular phylogenetic analysis of the FIV Vif and feline A3Z3.

Molecular phylogenetic analyses were performed as previously described (22, 24, 36, 54). Briefly, the sequences of FLV *vif* genes and feline *A3Z3* genes, some of which were newly identified in this study, were aligned using ClustalW implemented in MEGA7 (56). The MSA was verified manually at the amino acid level. Phylogenetic trees (Fig. 1 and 4) were reconstructed using the maximum likelihood (ML) method with PhyML (57).

### *dN/dS* analyses.

Diversifying selection sites in feline *A3Z3* genes were detected by FEL (37) and MEME (38), both of which are implemented in the Datamonkey website (http://www.datamonkey.org/).

### Plasmid construction.

The expression plasmids for HA-tagged domestic cat A3Z3 (hap I) (58), puma A3Z3 (23), lynx A3Z3 (23), and lion A3Z3 (23) were kindly provided by Carsten Münk (Heinrich-Heine-Universität, Düsseldorf, Germany). The expression plasmids for HA-tagged domestic cat A3Z3 (hap II and V) (22), bobcat A3Z3 (36), and cheetah A3Z3 (36) were prepared in our previous study. The expression plasmids for the HA-tagged A3Z3s of the other felids (listed in Table 1) were constructed by overlap extension PCR as previously described (36). The HA-tagged expression plasmids for domestic cat A3Z3 hap I A65R, domestic cat A3Z3 hap I L66R, domestic cat A3Z3 hap II L66R, ocelot A3Z3 R65A, ocelot A3Z3 R65S, and Asian golden cat A3Z3 R66L were constructed by overlap extension PCR as previously described (36). Briefly, each wild-type plasmid was used as the template, and the following primers were used (underlined nucleotides denote changes): domestic cat A3Z3 hap I A65R fwd, 5′-GAC AAG ATC AAG AGA CTG ACG CGG GAC ACA TCC CAG AGA TTC-3′; domestic cat A3Z3 hap I A65R rev, 5′-GAA TCT CTG GGA TGT GTC CCG CGT CAG TCT CTT GAT CTT GTC-3′; domestic cat A3Z3 hap I L66R fwd, 5′-GAC AAG ATC AAG GCA AGG ACG CGG GAC ACA TCC CAG AGA TTC-3′; domestic cat A3Z3 hap I L66R rev, 5′-GAA TCT CTG GGA TGT GTC CCG CGT CCT TGC CTT GAT CTT GTC-3′; domestic cat A3Z3 hap II L66R fwd, 5′-GAC AAG ATC AAG TCA AGG ACG CGG GAC ACA TCC CAG AGA TTC-3′; domestic cat A3Z3 hap II L66R rev, 5′-GAA TCT CTG GGA TGT GTC CCG CGT CCT TGA CTT GAT CTT GTC-3′; ocelot A3Z3 R65A fwd, 5′-GAC AAG ATC AAG GCC CTG ACG CGG GAC AAA-3′; ocelot A3Z3 R65A rev, 5′-TTT GTC CCG CGT CAG GGC CTT GAT CTT GTC-3′; ocelot A3Z3 R65S fwd, 5′-GAC AAG ATC AAG AGC CTG ACG CGG GAC AAA-3′; ocelot A3Z3 R65S rev, 5′-TTT GTC CCG CGT CAG GCT CTT GAT CTT GTC-3′; Asian golden cat A3Z3 R66L fwd, 5′-GAC AAG ATC AAG TCA CTG ACG CGG GAC ACA TC-3′; and Asian golden cat A3Z3 R66L rev, 5′-GAT GTG TCC CGC GTC AGT GAC TTG ATC TTG TC-3′.

The expression plasmids for His-tagged FIVfca Vif (strains Petaluma and TM2) (22, 24), FIVpco Vif (strains PLV1695 and Pco5), and FIVlru Vif (strain Lru1) (36) were prepared in our previous studies. To construct the expression plasmids for His-tagged FIVoma Vif (strain Oma3), FIVple Vif (strain 1027), and FIVppa Vif (strain Rasheed), the open reading frames (ORFs) were obtained from GeneArt gene synthesis service (Thermo Fisher Scientific). The obtained DNA fragments were digested with BamHI and SalI and inserted into the BamHI-SalI site of the pDON-AI plasmid (TaKaRa). The nucleotide sequences were determined by a DNA sequencing service (Fasmac, Kanagawa, Japan), and the data were analyzed by Sequencher v5.1 software (Gene Codes Corporation).

### Cell culture and transfection.

HEK293T cells (CRL-11268; ATCC) were cultured in Dulbecco’s modified Eagle medium (Sigma-Aldrich) supplemented with 10% heat-inactivated fetal calf serum and antibiotics (Thermo Fisher Scientific). Transfection was performed by using PEI Max (GE Healthcare) in accordance with the manufacturer’s procedures and described previously (10, 13, 22, 24, 36, 40, 42, 59–68). To analyze the dose-dependent anti-FIV activity of feline A3Z3, pFP93 (pFIVgagpolΔvif; a replication incompetent *vif*-deficient FIV packaging construct derived from clone FIV-34TF10 [GenBank accession number M25381]; kindly provided by Eric M. Poeschla) (200 ng), pTiger-luc (pFIVΨ-luc) (150 ng), and pMD.G (pVSVg; a vesicular stomatitis virus G [VSVg] expression plasmid) (50 ng) were cotransfected into HEK293T cells (1 × 10^5^ cells) with feline A3Z3 expression plasmid (50, 100, or 200 ng). To analyze the functional relationship between feline A3Z3 and FIV Vif, feline A3Z3 expression plasmid (200 ng), pFP93 (200 ng), pTiger-luc (150 ng), and pMD.G (50 ng) were cotransfected into HEK293T cells with or without His-tagged FIV Vif expression plasmid (400 ng). At 48 h posttransfection, the transfected cells and culture supernatants were harvested as previously described (22, 24, 36, 62).

### Western blotting.

Western blotting was performed as previously described (22, 24, 36, 62). For the Western blotting of virus particles, 340 μl of the culture supernatant was ultracentrifuged at 100,000 × *g* for 1 h at 4°C using a TL-100 instrument (Beckman), and the pellet was lysed with 1× SDS buffer. For the Western blotting of transfected cells, the cells were lysed with RIPA buffer (50 mM Tris-HCl buffer [pH 7.6], 150 mM NaCl, 1% Nonidet P-40, 0.5% sodium deoxycholate, 0.1% SDS) with protease inhibitor cocktail (Roche). The following antibodies were used for Western blotting: anti-His polyclonal antibody (OGHis; Medical and Biological Laboratories), anti-HA antibody (3F10; Roche), anti-FIV p24 capsid antibody (PAK3-2C1; Santa Cruz Biotechnology); and anti-alpha-tubulin (TUBA) antibody (DM1A; Sigma).

### FIV reporter assay.

FIV reporter assay was performed as previously described (22, 24, 36, 62). Briefly, 10 μl of the culture supernatant of transfected cells was inoculated into HEK293T cells in a 96-well plate (Nunc). Firefly luciferase activity was measured by using the BrillianStar-LT assay system (Toyo-b-net) and the 2030 ARVO X multilabel counter instrument (PerkinElmer) according to the manufacturers’ procedures.

### Protein homology modeling.

The protein homology model of domestic cat A3Z3 hap I protein was constructed in our previous study (22). The residues under diversifying selections as well as the residue 65 were plotted using PyMOL (PyMOL Molecular Graphics System, version 1.8; Schrödinger, LLC).

### Statistical analyses.

The data are expressed as averages with the standard errors of the means (SEMs), and statistically significant differences were determined by Student’s *t* test. In Fig. 3F and G, Spearman’s rank correlation coefficient was applied.

### Data availability.

The sequences of 11 feline *APOBEC3Z3* genes and FIVppa *vif* gene are available under GenBank/EMBL/DDBJ accession numbers LC597235 to LC597245 and LC599586.
